# An Overview of UBTF Neuroregression Syndrome

**DOI:** 10.3390/brainsci14020179

**Published:** 2024-02-15

**Authors:** Anneliesse A. Braden, Jianfeng Xiao, Roderick Hori, Chester Brown, Mohammad Moshahid Khan

**Affiliations:** 1 Department of Neurology, College of Medicine, University of Tennessee Health Science Center, Memphis, TN 38104, USA; abrade11@uthsc.edu (A.A.B.); jxiao@uthsc.edu (J.X.); 2 Neuroscience Institute, University of Tennessee Health Science Center, Memphis, TN 38163, USA; 3 Department of Microbiology, Immunology and Biochemistry, University of Tennessee Health Science Center, Memphis, TN 38163, USA; rhori@uthsc.edu; 4 Department of Pediatrics, College of Medicine, University of Tennessee Health Science Center, Memphis, TN 38163, USA; cbrow171@uthsc.edu; 5 Division of Regenerative and Rehabilitation Sciences, Department of Physical Therapy, Center for Muscle, Metabolism and Neuropathology, College of Health Professions, University of Tennessee Health Science Center, Memphis, TN 38163, USA

**Keywords:** childhood neurological disorders, neurodevelopment, neurodegeneration, UBTF, DNA damage response, behavioral symptoms

## Abstract

Recently, a recurrent de novo dominant mutation in *UBTF* (c.628G>A, p.Glu210Lys; *UBTF* E210K) was identified as the cause of a neurological disorder which has been named UBTF Neuroregression Syndrome (UNS), or Childhood-Onset Neurodegeneration with Brain Atrophy (CONDBA). To date, only 17 cases have been reported worldwide. The molecular etiology is a pathogenic variant, E210K, within the HMG-box 2 of Upstream Binding Transcription Factor (UBTF). UBTF, a nucleolar protein, plays an important role in ribosomal RNA (rRNA) synthesis, nucleolar integrity, and cell survival. This variant causes unstable preinitiation complexes to form, resulting in altered rDNA chromatin structures, rRNA dysregulation, DNA damage, and ultimately, neurodegeneration. Defining clinical characteristics of the disorder include but are not limited to developmental regression beginning at approximately three years of age, progressive motor dysfunction, declining cognition, ambulatory loss, and behavioral problems. Histological and neuroimaging abnormalities include cortical atrophy, white matter deficits, and enlarged ventricles. Herein, we present a detailed overview of all published cases as well as the functional roles of UBTF to better understand the pathophysiology. Bringing undiagnosed cases to the attention of clinicians and researchers by making them aware of the clinical features will improve research and support the development of therapeutic interventions.

## 1. Introduction

UBTF Neuroregression Syndrome (UNS), also known as Childhood-Onset Neurodegeneration with Brain Atrophy (CONDBA), is a neurodegenerative disease with first symptoms occurring at approximately age 3. UNS begins with the loss of developmental milestones, including motor and cognitive skills and sensorimotor function, progressing to pyramidal and extrapyramidal signs of neurological dysfunction, dysarthria, gait ataxia, ambulatory loss, seizures, dysphasia progressing to aphasia, and behavioral issues as seen in autism spectrum disorder. Brain anatomy is progressively affected. In general, the following pathologies develop: supratentorial cerebral atrophy, cerebellar atrophy, thinning of the corpus callosum, symmetric and diffuse white matter T2 hyperintensities, and ex vacuo ventriculomegaly. To our knowledge, there are only 17 reported patients worldwide [1,2,3,4,5,6,7].

The disease is caused by a recurrent de novo heterozygous, pathogenic variant in *UBTF* (c.628G>A, p.Glu210Lys) on chromosome 17 [1,2]. At the cellular level, this variant results in increased pre-rRNA levels, DNA damage, and apoptosis in fibroblasts from affected individuals, processes that are proposed to contribute to the delayed intellectual and behavioral development in UNS patients. Upstream Binding Transcription Factor (UBTF), a nucleolar protein, plays an important role in ribosomal RNA (rRNA) synthesis, nucleolar integrity, and cell survival. It belongs to a class of proteins containing nonspecific high-mobility group (HMG) boxes that contribute to DNA bending. UBTF has two isoforms, UBTF1 and UBTF2. UBTF1 facilitates the recruitment of RNA polymerase I (Pol I) for ribosomal DNA (rDNA) transcription and is integral in ribosome biogenesis [1,8]. The silencing of the Pol I co-factor, TIF-IA, attenuates rRNA transcription, causing nucleolar stress and p53-dependent neuronal apoptosis [9]. Impaired rRNA transcription and altered nucleolar integrity have been demonstrated in the brains of patients with neurodegeneration and neurodevelopmental disorders and in mouse models of general neurological disorders [10,11]. In contrast to UBTF1, UBTF2 regulates mRNA transcription through its effect on RNA polymerase II. The pathophysiology of UNS remains unclear, and there are no known disease-modifying interventions for this disease. In this article, we discuss recent progress in UNS research and highlight the molecular and clinical phenotypes of UNS patients and the approaches to improve clinical diagnosis.

## 2. Clinical Presentation

**Figure 1:**
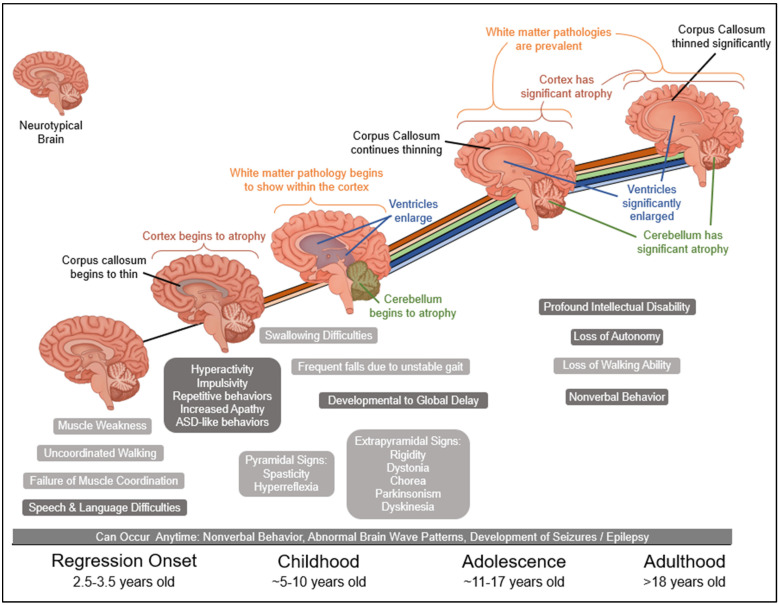
Generalized disease progression over time with brain illustrations. Illustration of a brain with UBTF Neuroregression Syndrome progressively aging and succumbing to the disease. The anatomical pathologies are shown in the illustrations and detailed above the curve. As an anatomical change occurs, a color-coded line is added to the timeline to show the compounding anatomical effects. For conceptualization, a neurotypical brain is shown in the top left of the figure. Below the curve, the physical and cognitive phenotypes are listed approximately around the time they would occur. The behavioral/cognitive symptoms have a slightly darker background than the physical symptoms. The wording of most phenotypes has been changed to more layman’s terms for ease of understanding, The proper medical terminology is used in the text.

Currently, the only diagnostic criterion for UBTF Neuroregression Syndrome is the presence of a pathogenic UBTF variant in HMG-box 2. Usually, the child presents with behaviors reminiscent of dementia, losing normal developmental milestones. After ruling out other neurological causes, genetic testing is performed, and the variant is discovered. Regression usually occurs between the ages of 2 and 4 years, but later childhood onset has been reported [1,7]. Early signs are speech and language difficulties; gait ataxia; hypotonia; and behavioral and cognitive disorders such as hyperactivity, impulsivity, and repetitive behaviors [1,2,4,7]. Once regression begins, the behavioral, physical, and anatomical symptoms set in rapidly (Figure 1).

Consistent clinical features are microcephaly, reported in 75% of published cases, and extrapyramidal signs including rigidity, dystonia, chorea, Parkinsonism, and dyskinesia are present in 71%. Only 4 of 17 reported Parkinsonism, 1 dyskinesia, 3 rigidity, and 4 chorea (Table 1). Pyramidal signs are slightly more prevalent, with 76% of cases having spasticity (12 of 17) and/or hyperreflexia (3 of 17) [1,2,3,4,5,6,7]. Frequent falls develop due to gait instability, with 76% reporting ataxia [6]. A total of 73% have swallowing difficulties, with two cases not reporting this symptom [2,3,4]. Dysarthria is frequently reported. Nine of the seventeen cases did not report dysarthria, perhaps because the syndrome is not well recognized, difficult to diagnose, and quite rare, and the definitive clinical features are still emerging, with many being non-specific. When dysarthria was considered in UNS case reports, in 8 of the 17 cases, 7 reported the presence of the symptom (Table 1). In combination with imaging and molecular testing, the characteristics listed above may be useful to establish a UNS diagnosis.

Cognitive abilities decline and the children typically become nonverbal. Challenging behavior emulating that of autism spectrum disorder (ASD) continues to worsen [7,12]. There are numerous accounts of progressive apathy [2,3,4]. By early adolescence, profound intellectual disability is present in 100% of reported patients. Eventually, there is a loss of ambulation and almost total loss of autonomy [1,2,3,4,5,6,7]. Abnormal EEG findings or seizures can develop at any time. There is inconclusive evidence linking the *UBTF* E210K variant to epilepsy, but the altered protein could be responsible for the atypical EEG findings [4,6]. Although the abnormal EEG findings cannot be definitively linked to the pathogenic *UBTF* variant, they might serve as an important diagnostic clue.

Brain magnetic resonance imaging (MRI) reveals atrophy of the supratentorial cerebrum and symmetric and/or diffuse white matter T2 hyperintensities in all cases. Most exhibit ventriculomegaly due to ex vacuo dilation of the ventricles and thinning of the corpus callosum. Some have cerebellar atrophy (Table 2). The findings vary in severity, but all pathologies progress with age [1,2,4,5,6,7]. Thalamic involvement with bilaterally symmetric hyperintensities in the ventromedial and dorsal thalamus has also been described [3]. Studying the thalamus may provide additional insight into the pathophysiology of the disease. In summary, the early signs include a loss of developmental milestones, speech and language difficulties, developmental delay, and autism-like behaviors. Other diagnostic clues include increased falls and gait instability, lack of muscle coordination, difficulty swallowing, and microcephaly. The characteristics of these patients exhibit considerable clinical overlap with several other neurologic conditions with a similar progression. The most helpful diagnostic tools are likely neuroimaging, perhaps EEG, and broad genetic testing that includes this gene in a larger panel, such as whole-exome or whole-genome analysis.

The level of severity with which a case will present is only found through the display of symptoms. The reason why one variation is able to result in phenotypes of differing intensities is unknown for UBTF syndrome. Table 1 provides an overview of all reported symptoms for all known cases by column. Cases with a heavy symptomatic burden can be easily identified by the number of listed phenotypes. These cases are in contrast with patients who have decreased symptom numbers. It is important to remember that UBTF syndrome is a complex and variable disorder. There is no single way to predict how severe a case will be, and even patients with few symptoms can experience significant challenges. This does not necessarily mean that a patient with fewer symptoms has a lower risk of developing serious complications. Each symptom can place a significant burden on patients and their families, and the absence of certain symptoms does not always indicate a milder course of the disease. The severity of individual symptoms can vary. Some symptoms of UBTF syndrome, such as seizures or intellectual disability, can be very debilitating even if they are the only symptoms present. Additionally, there may be hidden symptoms that have not yet been identified. As our understanding of UBTF syndrome improves, we may discover new symptoms that were previously overlooked.

## 3. Seizure Etiology and Abnormal Waveforms

Seizure activity is reported in 35% of patients with UBTF Neuroregression Syndrome. A total of 12% have abnormal EEG results but no observable seizures. Others have proposed that abnormal EEG waveforms could plausibly be due to the E210K variant, with onset corresponding to a specific stage of disease progression, but further investigation is needed [4]. However, 53% of patients have neither seizure activity nor abnormal EEG recordings [1,2,3,4,5,6,7]. We propose that seizures could be triggered indirectly due to UBTF-induced atrophic tissue, as cerebral atrophy can induce seizure activity. Additionally, UBTF cortical neurons have increased Na^+^ currents, synaptic amplitude, and hyperexcitability early in differentiation, as seen in patient-derived cortical neuron studies [12]. The hyperexcitability phenotype in cortical neurons is seen in a variety of ASD-related disorders, indicating that the individual genetic variation may not be at the root of the observed seizure activity [12]. With these data in mind, the presence of seizure activity may not be a beneficial guide for diagnosticians. While the presence of seizure activity can be a helpful indicator for diagnosing, it is not always reliable. In some cases, for instance, in 65% of known reports, seizure activity may not present itself, making diagnosis more challenging. However, once more cases have been identified and further research has been conducted, abnormal waveforms or even a specific pattern within EEG results could function as an indicator of UNS.

## 4. Molecular Genetics and Protein Function

**Figure 2:**
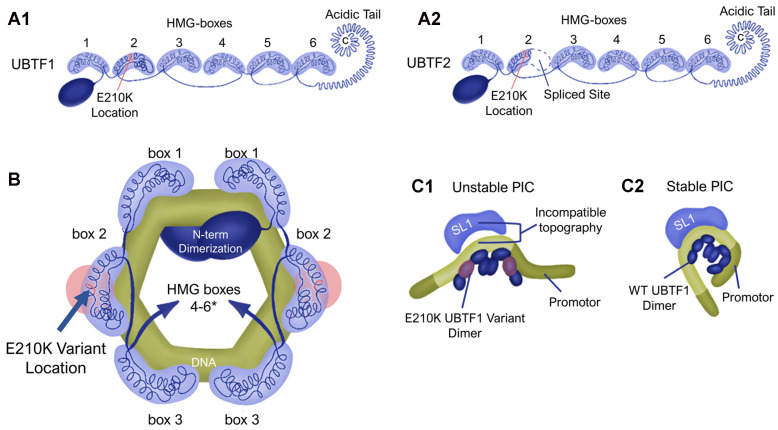
UBTF protein diagrams. (**A**) The 2D protein structure of both UBTF1 and UBTF2. (**A1**) UBTF1 protein diagram with E210K’s location marked. (**A2**) UBTF2 protein diagram with splicing location. The E210K variant still occurs in UBTF2, but the DNA bending ability has been lost, so the presence of E210K does not affect UBTF2. (**B**) UBTF functions as a dimer with the first 3 HMG-boxes bending ~140 bp of DNA into a 360-degree loop. (**C**) Preinitiation complex formation when there is a (**C1**) E210K variant and (**C2**) wild-type PIC. * Presumed location of HMG boxes 4–6.

UBTF is integral to ribosomal RNA synthesis and ribosome biogenesis. It functions as a dimer with six HMG-box domains, creating a 360° loop structure (Figure 2C) [1,8]. This physical structure enables UBTF to replace nucleosomes in the epigenetic remodeling of rDNA chromatin [8]. When examining the protein, it is easy to see that UBTF2 is shorter. UBTF2 is a splice variant missing 37 residues, resulting in a shorter HMG-box 2 than UBTF1, and, thus, does not have the HMG-box structure to recognize bent DNA (Figure 2A). In contrast, UBTF1 is able to bend the promotor, which creates the better recruitment of Selectivity Factor 1 (SL1) to generate a stable preinitiation complex (PIC) [13,14]. Only UBTF1 cooperates properly with SL1 to produce the PIC (Figure 2B). UBTF2 is not a part of the PIC, irrespective of the presence of UBTF1. This is consistent with an “induced fit” model, with UBTF1 being able to produce the proper binding site for SL1 [8,13]. Wild-type UBTF contains atypical residues in the central hydrophobic core of HMG-box 2, making this box less aromatic and establishing instability in this area [8]. In the variant E210K (Glu210Lys), the change from glutamic acid to lysine generates a highly positively charged stretch containing three consecutive lysine residues (Lys210-Lys211-Lys212) in the HMG-box 2 region. Consequently, the protein becomes a “hyperactive transcription factor” over-producing 18S rRNA, resulting in larger but fewer nucleoli per cell [1,8]. The E210K variant may further destabilize HMG-box 2, thereby impacting its ability to induce the proper topology for SL1 binding [13,15]. This is how the UBTF E210K variant reduces preinitiation complex formation and alters rRNA synthesis. There are adverse effects on development, growth, and cellular homeostasis when rRNA synthesis is negatively affected [8,16,17]. Deficiencies in rDNA transcription result in ribosome assembly deficits, leading to a reduction in ribosome number and the generation of inactive ribosomes, consequently impeding protein synthesis [8,18]. Additionally, the nucleolar stress induced by rDNA instability is associated with transcriptional deficits [8]. Altered rDNA chromatin structures and rRNA dysregulation, which occur as a consequence of the E210K variant, are linked to childhood neurodegeneration [1]. This error in PIC development on the rDNA is proposed as the cause of UNS [15]. Thus, there is clear evidence linking the UBTF E210K variant to UBTF Neuroregression Syndrome [1,3,15].

Evidently, changes in UBTF expression are found in some neurologic disorders including Alzheimer’s disease (AD) and Parkinson’s disease [19,20]. UBTF expression is downregulated in AD, which indicates a relationship between UBTF and neurologic malfunction [20]. The downregulation of UBTF in AD may also lead to impaired RNA polymerase I activity, which is essential for ribosomal biogenesis [20]. UBTF expression is also reduced in PD, and this decrease in expression may be linked to the degeneration of dopaminergic neurons resulting in the Parkinsonian phenotype. In PD, UBTF downregulation may be involved in the accumulation of alpha-synuclein, a protein that is a major component of Lewy bodies, which are characteristic of the disease [19]. This suggests that UBTF plays an important role in normal brain function and that changes in its expression can contribute to the symptoms of neurological diseases. To further understand the mechanistic biology involved with UBTF, Hori et al. employed a mouse model of Ubtf haploinsufficiency (Ubtf^+/−^) [2,21]. The homozygous global UBTF deletion was embryonic lethal, indicating that UBTF is essential for embryogenesis and adult survival. The Ubtf^+/−^ mouse model showed progressive neurobehavioral, cognitive, and motor deficits that progressed with age [2,21]. These results provide more insight into UBTF biology in human diseases.

The disruption in UBTF expression does not indicate a variation in the genome of the protein. The changes in UBTF expression are not due to mutations in the UBTF gene itself, but rather to other factors that regulate its expression. To understand more about the different variations, it needs to be determined how many variants exist and if they influence UNS. There are 78 total variants of the UBTF protein reported in ClinVar. The pathologic nature is not fully understood for most [8]. One of these variants was described in detail by Tinker et al. in 2022 [22]. They described a seemingly more severe UBTF disease associated with the Q203R variant. There were clear signs of regression at 9 months of age. This variant occurs in HMG-box 2 of the UBTF protein, just as E210K does [15]. It is plausible that Q203R could function similarly to E210K and via the same mechanisms, but additional research is required.

Prior to the publication of this report, if the disease was not referred to as CONDBA, UBTF Neuroregression Syndrome was primarily called “UBTF E210K Neuroregression Syndrome”, referencing the specific genetic mutation associated with the majority of identified cases. In an effort to provide more inclusivity within the UBTF diagnosis, the authors suggest the removal of the specific variant, E210K, from the syndrome name. The new name reflects the understanding that the syndrome encompasses a broader spectrum of UBTF-related variants beyond just the E210K mutation. This shift promotes inclusivity for individuals with other relevant UBTF variations who may not fit the previous, narrower definition. It is crucial to understand that simply having a UBTF variation does not automatically translate to a UNS diagnosis. The overall presentation must match previously reported cases and the variation must be working in a similar mechanistic way to the E210K variant, such as the Q203R variant discussed previously. Clinical consistency is necessary. Patients with a possible UNS diagnosis must exhibit clinical features aligning with previously reported cases. These features encompass a range of neurodevelopmental and behavioral challenges including motor difficulties, cognitive impairments, and speech problems. The specific UBTF variation in question must have a comparable functional impact on the protein to the well-studied E210K variant. For instance, the Q203R variant is currently recognized as another causative mutation due to its similar mechanism. As of now, only the E210K and Q203R variations meet the criteria for a CONDBA diagnosis. Further research may identify additional UBTF mutations with similar effects, potentially expanding the diagnostic scope in the future. Adopting the new terminology encourages a more comprehensive approach to UBTF-related neurodevelopmental disorders. It fosters inclusivity for individuals with diverse UBTF mutations and emphasizes the importance of both clinical presentation and the specific mechanism of the variant when making a diagnosis. This shift serves as a valuable step towards a more accurate and inclusive understanding of this complex condition.

## 5. Potential Molecular Mechanisms Associated with UBTF Neuroregression Syndrome

**Figure 3:**
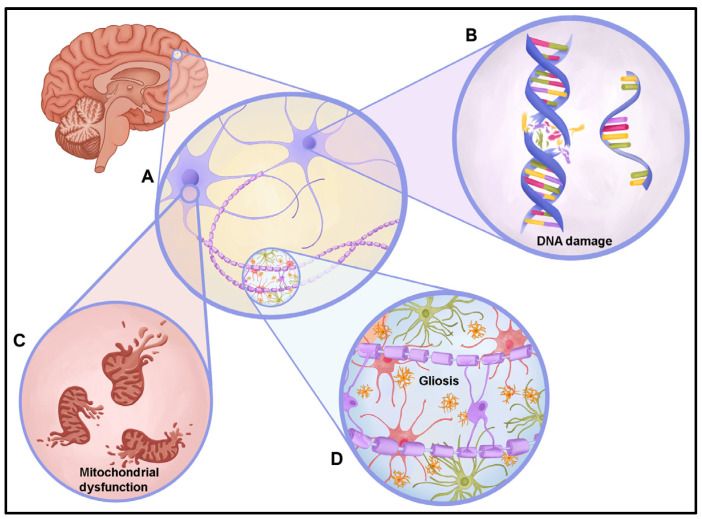
Possible pathologies of UNS illustration. An overall, simplified view of potential pathologies associated with UBTF Neuroregression Syndrome. (**A**) A close-up illustration of neurons with sections further deatail in the surrounding bubbles. (**B**) Double-stranded DNA damage and preRNA within the nucleus is represented. (**C**) Dysfunctional mitochindria are portrayed in the cytoplasm of the neuronal cell. (**D**) Excess glial cells (astrocytes, microglia, and oligodendrocytes) form gliosis depicted around axons.

The ultimate causes of neurodegeneration and behavioral deficits in UNS are unknown. Determining how a de novo mutation in *UBTF* impacts the UNS patient’s health and quality of life is a significant question, and neuronal function likely plays a critical role in these outcomes. Therefore, understanding the molecular and/or cellular mechanisms underlying neurobehavioral deficits will provide the foundation for novel therapeutic approaches that improve neurobehavioral functions in UNS patients. Based on observations to date, we surmise that ribosomal RNA transcriptional deficits and increased DNA damage have a causal role in the resulting pathologies (See Figure 3). UBTF plays a major role in DNA bending of the preinitiation complex in the ribosome biogenesis pathway. The E210K variant disrupts this essential DNA-bending function, resulting in unstable preinitiation complexes within the nucleolus [2,8,15]. Several nucleolar proteins are involved in DNA damage response (DDR). Consistent with these notions, genomic instability and DNA damage response have been associated with abnormal UBTF deficiencies [21,23]. The observed accumulation of DNA double-stranded breaks (DNA DSBs) in UNS-derived cells provides evidence that the DDR pathway is impacted [24,25].

DNA DSBs are the most harmful type of DNA damage. Impaired DNA DSB repair markedly impacts genome integrity, transcriptional activity, and neuronal/synaptic function [26,27,28].This is not unexpected as neurons are highly susceptible to the accumulation of DNA damage due to their high energy demand, increased transcriptional activity, and long lifespan. If the DNA damage remains unrepaired, signaling pathways downstream of DDR are activated, including mitochondrial dysfunction and other deleterious cell death pathways, all of which can lead to neurodevelopmental concerns and neurodegeneration.

DNA DSBs have a greater impact on cellular function and, thus, are often associated with neurodevelopmental defects such as microcephaly, e.g., Nijmegen breakage syndrome (mutated in NBS1), and ataxia–telangiectasia-like disorder (ATLD; mutated in MRE11). This is consistent with UNS in which UBTF patient-derived fibroblasts display an increased expression of DNA DSBs and increased nucleolar abnormalities [2]. Based on this observation, it is reasonable to infer that the E210K mutation in UBTF has a greater impact on DDR and suggests that targeting DDR mechanisms may hold therapeutic promise.

A possible secondary effect of unrepaired DNA damage and the resulting disruption of or decrease in DNA repair pathways in the nucleus is mitochondrial dysfunction. Unrepaired DNA damage in the nucleus can trigger a cascade of detrimental effects, including the disruption or downregulation of DNA repair pathways. All but 13 mitochondrial genes are transcribed in the nucleus, so when excessive nuclear DNA damage occurs, mitochondrial dysfunction can be expected. This compromised nuclear environment critically impacts mitochondria. Disruptions in nuclear DNA repair machinery can therefore ripple down to affect mitochondrial function [29,30]. Essentially, nuclear turmoil spills over, impacting the very source of cellular energy. A protein named peroxisome proliferator-activated receptor gamma coactivator 1-alpha (PPARGC1A), known as a “master regulator” of mitochondrial biogenesis, experiences a compensatory upregulation when UBTF is knocked down in 3T3 cells [2]. This suggests that UBTF dysfunction may trigger an attempt by the cell to increase mitochondrial production as a response to stress. A complementary upregulation of PPARGC1A was indicated in UBTF E210K patient-derived fibroblasts, further solidifying the potential link [2]. Similarly, in the only UBTF patient muscle biopsy report (n of 2), compensatory mitochondrial proliferation was seen via subsarcolemmal rims in cytochrome histochemical activity, suggesting that mitochondrial dysfunction plays a causative role in UNS pathology [7]. These data combined demonstrate that the underlying disease mechanism of UNS is potentially relevant to mitochondrial function and subsequent mitochondrial biogenesis (Figure 3). However, further studies are required to understand the molecular and cellular mechanisms underlying UNS. The complex interplay between nuclear DNA damage, UBTF function, and mitochondrial dysfunction in UBTF Neuroregression Syndrome (UNS) is an emerging area of research, offering potential insights into the underlying disease mechanisms.

## 6. Future Research Directions

While there has been much progress toward understanding the role of the UBTF E210K variant in UNS, a range of additional work is needed. The ultimate objective is early detection with prompt therapeutic intervention to either slow or halt disease progression. Awareness of this disorder is essential to improve diagnostic criteria for clinicians and provide advantageous early identification. Currently, there are 17 published cases. It is expected and reasonable to assume that additional children and parents are affected with this condition, with some going through the process of diagnostic testing for an unidentified disorder that will ultimately be diagnosed as UBTF E210K syndrome. These individuals and their cases are currently invisible to researchers. Since the rate of research progress and therapeutic interventions will increase as new cases are identified, it is important to elucidate molecular and cellular mechanisms to facilitate this process. Clinicians can help their patients by carrying out clinical genetic testing and sharing information about confirmed *UBTF* E210K cases to assist research initiatives. If physicians are unable to report in a timely manner, molecular diagnostic laboratories used by clinics and hospitals could share de-identified results reporting pathogenic *UBTF* mutations, accessible to clinicians and the research community. The same idea applies to other variants of *UBTF*, particularly Q203R. Physicians and scientists lack knowledge about this specific variant. The use of patient-derived stem cells would allow researchers to provide an individualized approach to patient care. Researchers will expand their assortment of patient-derived stem cells when additional patients are identified. While these pieces of evidence paint a suggestive picture, a deeper understanding of the molecular and cellular mechanisms underlying the UBTF–mitochondrial dysfunction connection in UNS remains crucial. Future research should delve into the precise pathways by which UBTF dysfunction impacts mitochondrial biogenesis and function. Exploring the specific effects of different UBTF mutations on mitochondrial activity and exploring potential therapeutic interventions targeting this connection are additional crucial areas of exploration. Furthermore, this approach enables additional understanding of the biological mechanisms that underly pathological *UBTF* variants and their resulting phenotype. By deciphering these intricate connections, researchers can unlock new avenues for the diagnosis, treatment, and potentially even the prevention of UNS.

## 7. Conclusions and Perspectives

UBTF Neuroregression Syndrome (UNS), also known as Childhood-Onset Neurodegeneration with Brain Atrophy (CONDBA), is a devastating diagnosis for a family to navigate and is difficult to diagnose for clinicians. Supplying stakeholders with as much knowledge as possible is beneficial for all. There is early onset, at around 3 years of age, with the loss of developmental milestones. Physical limitations develop quickly, as does cognitive decline. Behavioral disorders with qualities akin to autism spectrum disorder are also evident. The brain develops anatomical pathologies, most notably cerebral atrophy and white matter tract deformities, leading to advanced debilities. Studying the thalamus may provide additional insights into the pathophysiology of the disease. Based on current knowledge and observations to date, by adolescence, affected individuals are nonverbal, non-ambulatory, and reliant on a feeding tube with a severe intellectual disability and a complete loss of autonomy. Currently, 17 cases of UBTF Neuroregression Syndrome are known worldwide, but there are certainly additional cases unrevealed to researchers. A better understanding of UNS will help identify novel potential therapeutic and diagnostic tools for this disease. The establishment of unified UNS diagnostic criteria will help physicians to identify the disorder in a faster time span. Early detection is essential for potential pharmaceutical interventions. To develop a therapeutic approach, we must elucidate how UBTFE210K drives cellular dysfunction, which is ultimately linked with behavioral and intellectual disabilities and neurodegenerative changes. Future research should delve into the specific pathways by which UBTF dysfunction impacts DNA damage response, mitochondrial biogenesis, function, and communication with other cellular components. Understanding these intricate threads is crucial to untangling the disease web. In recent years, small molecules targeting rRNA dysregulation, a potential consequence of UBTF dysfunction, or the DNA damage response pathway have been explored in disease models, showing promising initial results. Oligonucleotide-based therapeutics designed to interact with specific RNA sequences, which show potential in correcting gene expression imbalances, have been used in disease models, offering another possible avenue for intervention. While the full picture of UBTF’s role in UNS remains under investigation, the ongoing research paints a hopeful future. By unraveling the intricate molecular mechanisms at play, researchers can unlock new avenues for diagnosis, treatment, and potentially even the prevention of UNS and related disorders. This will ultimately lead to a future in which individuals with UNS can live healthier, more fulfilling lives.

## Figures and Tables

**Table 1:** Detailed phenotypes of UBTF E210K patients by article. Each column details the reported phenotypes of one patient as well as demographic information. If an area is blank, then the article did not explicitly indicate this symptom for the patient. The average age of regression is 3.5 years. NA descibes when the article did not specifiy information about this symptom.

Reference	Edvardson, 2017 [1]							Toro, 2018 [2]				Sedlackova, 2018 [3]	Bastos, 2020 [4]	Ikeda, 2021 [5]	Gunasekaran, 2023 [6]	Pietra, 2023 [7]	
Total reported patients	7							4				1	1	1	1	2	
Gender	F	F	F	F	F	M	F	M	M	F	F	M	M	F	M	M	F
Age at publication	23	17	16	19	19	11	8	12	6.2	33	12	13	12	19	10	16	22
Age at regression	2.5	5	7	3	3	4	3.5	2.5	2.25	3	3	2	5.8	2	3	6	2
Country of origin/ethnicity	USA	Canada	USA	France	Israel	Russia	USA	Caucasian, European	Ashkenazi Jewish	Caucasian, European	Caucasian, European	Czechia	Sri Lanka	Japan	India	NA	NA
Phenotype																	
Developmental delay prior to regression		DD prior to regression	DD prior to regression				DD prior to regression					Speech delay prior to regression			DD prior to regression		
Microcephaly	MC	MC	MC	MC	MC	MC		MC	MC	NA	NA	NA			MC	MC	MC
Ataxia	Ataxia		Ataxia				Ataxia	Ataxia	Ataxia	Ataxia	Ataxia	Ataxia	Ataxia	Ataxia	Ataxia	Ataxia	Ataxia
Hypotonia	NA	NA	NA	NA	NA	NA	NA	Hypotonia	Hypotonia		Hypotonia		Hypotonia				
Extrapyramidal signs	Dystonia	Dystonia, rigidity		Dystonia, chorea	Parkinsonism	Dystonia		Dystonia, chorea		Dystonia	Dystonia	Dystonia	Dystonia, chorea	Dystonia, Parkinsonism, Dyskinesia, rigidity, chorea		Dystonia, Parkinsonism	Dystonia, Parkinsonism, rigidity
Pyramidal signs		Spasticity	Spasticity	Spasticity	Spasticity	Spasticity	Spasticity	Spasticity	Spasticity, hyperreflexia	Spasticity, hyperreflexia	Spasticity	Spasticity, hyperreflexia	bDTR		bDTR, spasticity		
Behavioral disorders	NA	NA	NA	NA	NA	NA	NA	Autistic, mildly self-injurious, not fully toilet trained, inattentive	Mouthing of hands	Compulsive, repetitive, distractible, hyperactive	Exhibited parallel play but not group play	Apathy, social interaction difficulties	Apathy, agitation, friendliness	NA	NA	Mildly self-injurious, compulsive–obsessive	Irritability, no social interaction
Dysarthria	NA	NA	NA	NA	NA	NA	NA	Dysarthria	Dysarthria	Dysarthria	Dysarthria	NA	Dysarthria	Dysarthria	NA	Dysarthria	Dysarthria
Swallowing difficulties/ dysphagia	5 of 7 cases, not reported individually							Swallowing difficulties		Swallowing difficulties, feeding tube	Swallowing difficulties, feeding tube	Swallowing difficulties	Swallowing difficulties	NA	NA		Swallowing difficulties, feeding tube
Seizures		Seizures	Seizures	Seizures								Severe Seizures	Seizures		Seizures		
Abnormal EEG						Abnormal EEG								Abnormal EEG			
Outcome	Non-ambulatory, nonverbal	Non-ambulatory, nonverbal	Non-ambulatory, nonverbal	Non-ambulatory, nonverbal	Non-ambulatory, nonverbal	Non-ambulatory, nonverbal	Assisted walking			Walked few steps, nonverbal		Non-ambulatory, nonverbal	Non-ambulatory, Nonverbal	Ventilatory support			Non-ambulatory, ventilatory support, nonverbal
Intellectual disability	ID	ID	ID	ID	ID	ID	ID	Severe ID	Severe ID	Severe ID	Severe ID	Severe ID	Severe ID	ID	ID	ID	ID

Abbriviations: DD, developmental delay; MC, microcephaly; ID, intellectual disability; EEG, electroencephalogram; NA, not available.

**Table 2:** Detailed anatomical pathologies of UBTF E210K patients by article. Each column details the reported physical anomolies of one patient as well as demographic information. Of note, every report indicated the presence of cerebral atrophy and white matter hyperintensities. If an area is blank, then the article did not explicitly indicate this symptom for the patient. NA descibes when the article did not specifiy information about this symptom.

Reference	Edvardson, 2017 [1]							Toro, 2018 [2]				Sedlackova, 2018 [3]	Bastos, 2020 [4]	Ikeda, 2021 [5]	Gunasekaran, 2023 [6]	Pietra, 2023 [7]	
Total reported patients	7							4				1	1	1	1	2	
Gender	F	F	F	F	F	M	F	M	M	F	F	M	M	F	M	M	F
Age at publication	23	17	16	19	19	11	8	12	6.2	33	12	13	12	19	10	16	22
Age at regression	2.5	5	7	3	3	4	3.5	2.5	2.25	3	3	2	5.8	2	3	6	2
Thinning of the corpus callosum	NA	Thinning of the CC	NA	NA	NA	NA	NA	Each case report thinning of the CC				NA	NA	Thinning of the CC	NA		Thinning of the CC
Cerebral atrophy	All known cases report cerebral atrophy																
Cerebellar atrophy	Cerebellar atrophy	Cerebellar atrophy	Cerebellar atrophy	Cerebellar atrophy			Cerebellar atrophy		Cerebellar atrophy	Cerebellar atrophy	Cerebellar atrophy		Cerebellar atrophy		Cerebellar atrophy		Cerebellar atrophy
Diffuse white matter hyper intensities	All known cases report white matter hyper intensities																
Ventriculomegaly/ex vacuo dilation of the ventricles	NA	Enlarged ventricles	NA	NA	NA	NA	NA	Each case reported enlarged ventricles					Enlarged ventricles		NA	NA	NA

Abbriviations: CC, corpus callosum; NA, not available.

## Data Availability

Not applicable.

## References

[1] Edvardson S., Nicolae C.M., Agrawal P.B., Mignot C., Payne K., Prasad A.N., Prasad C., Sadler L., Nava C., Mullen T.E. (2017). Heterozygous De Novo UBTF Gain-of-Function Variant Is Associated with Neurodegeneration in Childhood. Am. J. Hum. Genet..

[2] Toro C., Hori R.T., Malicdan M.C.V., Tifft C.J., Goldstein A., Gahl W.A., Adams D.R., Fauni H.B., Wolfe L.A., Xiao J. (2018). A Recurrent de Novo Missense Mutation in UBTF Causes Developmental Neuroregression. Hum. Mol. Genet..

[3] Sedláčková L., Laššuthová P., Štěrbová K., Haberlová J., Vyhnálková E., Neupauerová J., Staněk D., Šedivá M., Kršek P., Seeman P. (2019). UBTF Mutation Causes Complex Phenotype of Neurodegeneration and Severe Epilepsy in Childhood. Neuropediatrics.

[4] Bastos F., Quinodoz M., Addor M.-C., Royer-Bertrand B., Fodstad H., Rivolta C., Poloni C., Superti-Furga A., Roulet-Perez E., Lebon S. (2020). Childhood Neurodegeneration Associated with a Specific UBTF Variant: A New Case Report and Review of the Literature. BMC Neurol..

[5] Ikeda C., Kawarai T., Setoyama C., Orlacchio A., Imamura H. (2021). Recurrent de Novo Missense Variant E210K in UBTF Causes Juvenile Dystonia-Parkinsonism. Neurol. Sci..

[6] Gunasekaran P.K., Laxmi V., Manjunathan S., Kumar A., Tiwari S., Saini L. (2023). Childhood-Onset Neurodegeneration with Brain Atrophy in Association with c.628G>A in UBTF Gene. Indian. J. Pediatr..

[7] Pietra A., Palombo F., Giannotta M., Maffei M., Fiorini C., Costa R., Cenacchi G., Carelli V., Cordelli D.M., Pini A. (2023). Expanding the Clinical Spectrum of UBTF-Related Neurodevelopmental Disorder. Neurol. Genet..

[8] Moss T., LeDoux M.S., Crane-Robinson C. (2023). HMG-Boxes, Ribosomopathies and Neurodegenerative Disease. Front. Genet..

[9] Kalita K., Makonchuk D., Gomes C., Zheng J., Hetman M. (2008). Inhibition of Nucleolar Transcription as a Trigger for Neuronal Apoptosis. J. Neurochem..

[10] Parlato R., Kreiner G. (2013). Nucleolar Activity in Neurodegenerative Diseases: A Missing Piece of the Puzzle?. J. Mol. Med..

[11] Hetman M., Slomnicki L.P. (2019). Ribosomal Biogenesis as an Emerging Target of Neurodevelopmental Pathologies. J. Neurochem..

[12] Hussein Y., Tripathi U., Choudhary A., Nayak R., Peles D., Rosh I., Rabinski T., Djamus J., Vatine G.D., Spiegel R. (2023). Early Maturation and Hyperexcitability Is a Shared Phenotype of Cortical Neurons Derived from Different ASD-Associated Mutations. Transl. Psychiatry.

[13] Stefanovsky V.Y., Moss T. (2008). The Splice Variants of UBF Differentially Regulate RNA Polymerase I Transcription Elongation in Response to ERK Phosphorylation. Nucleic Acids Res..

[14] Friedrich J.K. (2005). TBP-TAF Complex SL1 Directs RNA Polymerase I Pre-Initiation Complex Formation and Stabilizes Upstream Binding Factor at the rDNA Promoter. J. Biol. Chem..

[15] Tremblay M.G., Sibai D.S., Valère M., Mars J.-C., Lessard F., Hori R.T., Khan M.M., Stefanovsky V.Y., LeDoux M.S., Moss T. (2022). Ribosomal DNA Promoter Recognition Is Determined in Vivo by Cooperation between UBTF1 and SL1 and Is Compromised in the UBTF-E210K Neuroregression Syndrome. PLoS Genet..

[16] Scott M., Klumpp S., Mateescu E.M., Hwa T. (2014). Emergence of Robust Growth Laws from Optimal Regulation of Ribosome Synthesis. Mol. Syst. Biol..

[17] Dai X., Zhu M. (2020). Coupling of Ribosome Synthesis and Translational Capacity with Cell Growth. Trends Biochem. Sci..

[18] Scull C.E., Schneider D.A. (2019). Coordinated Control of rRNA Processing by RNA Polymerase I. Trends Genet..

[19] Garcia-Esparcia P., Hernández-Ortega K., Koneti A., Gil L., Delgado-Morales R., Castaño E., Carmona M., Ferrer I. (2015). Altered Machinery of Protein Synthesis Is Region- and Stage-Dependent and Is Associated with α-Synuclein Oligomers in Parkinson’s Disease. Acta Neuropathol. Commun..

[20] Hernández-Ortega K., Garcia-Esparcia P., Gil L., Lucas J.J., Ferrer I. (2016). Altered Machinery of Protein Synthesis in Alzheimer’s: From the Nucleolus to the Ribosome. Brain Pathol..

[21] Hori R.T., Moshahid Khan M., Xiao J., Hargrove P.W., Moss T., LeDoux M.S. (2022). Behavioral and Molecular Effects of Ubtf Knockout and Knockdown in Mice. Brain Res..

[22] Tinker R.J., Guess T., Rinker D.C., Sheehan J.H., Lubarsky D., Porath B., Mosera M., Mayo P., Solem E., Lee L.A. (2022). A Novel, Likely Pathogenic Variant in UBTF-related Neurodegeneration with Brain Atrophy Is Associated with a Severe Divergent Neurodevelopmental Phenotype. Mol. Genet. Genom. Med..

[23] Sanij E., Diesch J., Lesmana A., Poortinga G., Hein N., Lidgerwood G., Cameron D.P., Ellul J., Goodall G.J., Wong L.H. (2015). A Novel Role for the Pol I Transcription Factor UBTF in Maintaining Genome Stability through the Regulation of Highly Transcribed Pol II Genes. Genome Res..

[24] Korsholm L.M., Gál Z., Nieto B., Quevedo O., Boukoura S., Lund C.C., Larsen D.H. (2020). Recent Advances in the Nucleolar Responses to DNA Double-Strand Breaks. Nucleic Acids Res..

[25] Ogawa L.M., Baserga S.J. (2017). Crosstalk between the Nucleolus and the DNA Damage Response. Mol. BioSyst..

[26] Dileep V., Boix C.A., Mathys H., Marco A., Welch G.M., Meharena H.S., Loon A., Jeloka R., Peng Z., Bennett D.A. (2023). Neuronal DNA Double-Strand Breaks Lead to Genome Structural Variations and 3D Genome Disruption in Neurodegeneration. Cell.

[27] Thadathil N., Hori R., Xiao J., Khan M.M. (2019). DNA Double-Strand Breaks: A Potential Therapeutic Target for Neurodegenerative Diseases. Chromosome Res..

[28] Welch G., Tsai L.-H. (2022). Mechanisms of DNA Damage-Mediated Neurotoxicity in Neurodegenerative Disease. EMBO Rep..

[29] Sharma N., Pasala M.S., Prakash A. (2019). Mitochondrial DNA: Epigenetics and Environment. Environ. Mol. Mutagen..

[30] Nadalutti C.A., Ayala-Peña S., Santos J.H. (2022). Mitochondrial DNA Damage as Driver of Cellular Outcomes. Am. J. Physiol. Cell Physiol..

